# Dietary interventions to improve body composition in men treated with androgen deprivation therapy for prostate cancer: a solution for the growing problem?

**DOI:** 10.1038/s41391-021-00411-7

**Published:** 2021-06-30

**Authors:** Lisa Umlauff, Manuel Weber, Nils Freitag, Ciaran M. Fairman, Axel Heidenreich, Wilhelm Bloch, Moritz Schumann

**Affiliations:** 1 Department for Molecular and Cellular Sports Medicine, Institute of Cardiovascular Research and Sports Medicine, German Sport University Cologne, Cologne, Germany; 2 Olympic Training Centre Berlin, Berlin, Germany; 3 Department of Exercise Science, University of South Carolina, Columbia, SC USA; 4 Department of Urology, University Hospital Cologne, Cologne, Germany

**Keywords:** Prostate cancer, Cancer metabolism, Prostate cancer, Cancer therapy

## Abstract

**Background:**

Androgen deprivation therapy (ADT) has adverse effects on body composition, including muscle wasting and body fat accumulation, which may be attenuated by nutrition therapy. This systematic review summarises available evidence on the effects of dietary interventions on lean mass, fat mass and body mass index (BMI) in men treated with ADT for prostate cancer.

**Methods:**

MEDLINE, Embase, Web of Science and ClinicalTrials.org were searched from inception through December 2020. We included all controlled trials evaluating effects of supplementation or dietary interventions on body composition in men with prostate cancer receiving continuous ADT. Methodological quality of the studies was assessed using the Cochrane Collaboration’s risk of bias tool. Meta-analysis was performed using a random effects model to calculate standardised mean differences between intervention and comparator groups. (PROSPERO; CRD42020185777).

**Results:**

Eleven studies (*n* = 536 participants) were included. Seven studies investigated the effects of dietary advice interventions, e.g. individual or group counselling, and four studies included a nutritional supplement. Eight studies combined the dietary intervention with exercise. Nine studies reported sufficient data for inclusion in the meta-analysis. Dietary advice and supplementation interventions combined were not associated with significant changes in lean mass (0.05 kg; 95% CI: −0.17, 0.26; *p* = 0.674; *n* = 355), fat mass (−0.22 kg; 95% CI: −0.45, 0.01; *p* = 0.064; *n* = 336) or BMI (−0.16 kg*m^−2^; 95% CI: −0.37, 0.04; *p* = 0.121; *n* = 399). Dietary advice interventions alone were associated with a significant fat mass reduction (−0.29 kg; 95% CI: −0.54, −0.03; *p* = 0.028; *n* = 266).

**Conclusions:**

Most studies were dietary advice interventions targeting caloric restriction, which showed the potential to reduce fat mass but did not increase lean mass in men treated with ADT. Future interventions should investigate whether a combination of dietary advice and protein supplementation with concomitant resistance exercise could counteract ADT-induced muscle wasting.

## Introduction

Prostate cancer is the second most commonly diagnosed cancer in men globally and its incidence is projected to rise as the world population ages [1, 2]. Androgen deprivation therapy (ADT) reduces prostate cancer growth and disease-specific mortality, and thus is considered the standard treatment for advanced prostate cancer [3]. However, the reduction of testosterone to castrate levels causes severe adverse effects, such as sexual dysfunction and fatigue, and is further associated with adverse changes in body composition, including reduced bone mineral density, increased fat mass and loss of skeletal muscle mass [4, 5]. In turn, long-term treatment with ADT is associated with a higher risk for developing metabolic syndrome, osteoporosis and cardiovascular disease [6–8]. Moreover, these body composition changes are often accompanied by a reduction in muscle strength, physical function and quality of life [9, 10].

Age-related loss of skeletal muscle mass and strength is associated with an increased risk for morbidity and mortality in the general population [11, 12]. In men treated with ADT, this ageing process is exacerbated due to suppression of testosterone, an anabolic steroid that promotes muscle growth [13] but is also involved in prostate cancer pathogenesis [14]. The accelerated deterioration of body composition is supported by studies showing a 2–4% decrease of lean mass and a concomitant 14% increase in fat mass in men with prostate cancer after 36 weeks on ADT, often resulting in sarcopenic obesity [5, 15]. Furthermore, ADT-induced lean mass changes appear to affect the limbs more than the trunk [5]. Exacerbated muscle wasting of the limbs may augment the decline in physical function and contribute to the loss of autonomy in men with advanced disease. Indeed, long-term ADT is associated with decreased biomechanical function of the lower-limb muscles during walking [16]. In addition, the risk of prostate cancer recurrence after primary treatment reportedly increases by 21% per 5 kg*m^−2^ growth in BMI, suggesting a higher risk of disease-specific mortality for patients with obesity [17]. In fact, higher values of both skeletal muscle mass and muscle density have been associated with a reduced mortality risk for men with advanced disease [18, 19]. As treatment with ADT might continue for several years, there is a growing need for interventions that alleviate the disease burden caused by prostate cancer and its treatment.

To treat catabolic alterations in patients with cancer, a combination of nutrition therapy and physical exercise is recommended [20]. While resistance exercise is well established as an effective strategy to promote muscle growth in healthy ageing adults [21], a previous meta-analysis found that in men treated with ADT supervised exercise interventions failed to induce changes in lean mass [22]. However, the authors purposefully excluded interventions with a diet component and argued that concomitant protein supplementation may increase lean mass in this population. Indeed, it has been demonstrated that protein intake can promote skeletal muscle growth and enhance the effects of resistance exercise in healthy individuals [23, 24] and patients with cancer [25]. Physiologically, the stimulation of muscle protein synthesis by dietary protein is driven by the increased availability of amino acids [24]. Increasing the dietary protein intake has shown to be an effective and inexpensive strategy to counteract age-related loss of skeletal muscle mass [24], and therefore may benefit men treated with ADT. Furthermore, dietary interventions aiming to decrease fat intake or restrict calories have been associated with body mass reductions in men with prostate cancer [26]. However, these results are not specific to men treated with ADT and caution is warranted as the interpretation of body mass alone, without accounting for body composition, may be misleading due to the complex metabolic state induced by ADT. Whether dietary interventions can attenuate or even reverse the adverse effects of ADT on body composition, specifically lean mass, remains therefore unclear.

The objective of the present systematic review and meta-analysis was to summarise the available evidence on the potential benefits of nutritional supplementation or dietary advice interventions for men treated with ADT for prostate cancer to counteract treatment-related changes in body composition, specifically muscle wasting and increased body fat.

## Methods

### Literature search

A systematic search of peer-reviewed literature was conducted in accordance with the Preferred Reporting Items for Systematic Reviews and Meta-Analyses (PRISMA) [27]. This project was registered in the international prospective register of systematic reviews (PROSPERO; CRD42020185777). The search included the databases MEDLINE, Embase and Web of Science, as well as the clinical trial register ClinicalTrials.gov. Databases were searched from their inception until December 21st, 2020. The search string included terms related to prostate cancer (e.g. prostatic tumour) and nutrition (e.g. diet, supplementation). The individual search strings for each database are presented in Table 1. No language restrictions were applied. In addition, reference lists of included studies and reviews were searched by hand for relevant studies.

**Table 1:** Search string for each database.

Database	Search string
MEDLINE	(prostatic neoplasms[MeSH Terms] OR (prostate cancer^*^[tiab]) OR (prostatic cancer*[tiab]) OR (prostate tumour*[tiab]) OR (prostate tumour*[tiab]) OR (prostate carcinoma*[tiab]) OR (prostate neoplasm*[tiab])) AND ((dietary supplements[MeSH Terms]) OR (nutrition therapy[MeSH Terms]) OR diet[MeSH Terms] OR (supplement*[tiab]) OR (diet[tiab]) OR (dietary[tiab]) OR (nutrition*[tiab]) OR (food*[tiab]) OR (nutrient*[tiab]) OR (nourishment[tiab]) OR (aliment*[tiab]) OR (lifestyle[tiab]))
Embase	(1) exp prostate tumour/ (2) (prostat* adj3 (cancer* or carcinoma* or malignan* or tumour* or tumour* or neoplas* or adenocarcinoma*)).tw,kw. (3) or/1–2 (4) dietary supplement/ (5) dietary supplementation/ (6) diet therapy/ (7) exp diet/ (8) (supplement* or diet or dietary or nutrition* or food* or nutrient* or nourishment or aliment* or lifestyle).tw,kw. (9) or/4–8 (10) 3 and 9 (11) limit 10 to embase
Web of Science	TS = (Prostat* AND (Cancer* OR tumo$r* OR carcinoma* OR neoplasm*) AND (diet* OR supplement* OR nutrition* OR food* OR nutrient* OR nourishment OR aliment* lifestyle OR feeding* OR nutriment*))
ClinicalTrials.gov	(1) Filter: prostate cancer [condition], androgen deprivation [other], supplementation [intervention], male [sex] (2) Filter: prostate cancer [condition], androgen deprivation [other], diet [intervention], male [sex]

### Study selection

Duplicate articles were removed using EndNote (version X9, Clarivate, Philadelphia, PA, USA). Two authors (LU, MW) independently screened the titles and abstracts of identified studies to assess eligibility. The full texts of relevant articles were retrieved and assessed for eligibility. Disagreements were resolved through consultation with a third author (NF).

#### Eligibility criteria

The inclusion criteria followed the PICOS (participants, interventions, comparisons, outcomes, study design) model [28]. Studies were eligible if they included: (P) men with clinically diagnosed prostate cancer who received any form of ADT (i.e. hormone therapy or bilateral orchiectomy) and continued the treatment for the duration of the study; (I) a dietary intervention component aimed to improve body composition outcomes, either a direct (e.g. nutritional supplementation) or indirect (e.g. nutrition advice) manipulation of dietary intake, regardless of a physical activity component; (C) a comparator group with either a placebo or no dietary intervention, regardless of a physical activity component; (O) an objective measure of body composition via direct (e.g. dual-energy X-ray absorptiometry (DXA)) or indirect (e.g. bioelectrical impedance analysis, anthropometry) methods, including at least one of the following outcomes: lean mass, fat mass or body mass index (BMI); (S) a controlled study design, either a randomised controlled trial (RCT) or controlled trial, with outcomes assessed pre and post intervention, i.e. no cross-sectional studies. Dietary interventions solely aimed at inhibiting cancer progress were excluded. Eligibility was irrespective of participants’ age, disease stage or concomitant treatments. Abstracts were eligible if sufficient information about the intervention was available from study protocols and the authors provided additional data.

### Data collection

Data from all included studies were extracted into a purposefully developed spreadsheet by two authors (LU, MW). Extracted data included: (1) general information (authors, year of publication, aim, study design); (2) participant information (sample size, age, type of ADT, time on ADT); (3) intervention details (intervention protocol, setting, duration); (4) findings (body composition outcomes, compliance, adverse events). Authors were contacted for further information or to clarify study procedures, if needed. If additional data was provided, it was included in the analysis [29–31].

### Risk of bias assessment

The Cochrane Collaboration’s tool for assessing risk of bias [32] was used to assess the methodological quality of the included studies. Two authors (LU, MW) independently examined the studies for the following potential sources of bias: selection (random sequence generation; allocation concealment), performance (blinding of participants and personnel), detection (blinding of outcome assessment), attrition (incomplete outcome data), reporting (selective reporting) and other biases (e.g. reporting of intervention design and adherence). Risk of bias was only assessed for full-text publications.

### Data analysis

The analyses were performed using R version 4.0.3 [33], RStudio [34] and the metafor package version 2.4.0 [35]. Post-intervention values of lean mass, fat mass and BMI of the included studies were pooled using a random effects model. If studies reported both absolute and relative body composition measures, only absolute measures were included in the analysis. Statistical heterogeneity (τ^2^) was assessed using the restricted maximum-likelihood estimator and, additionally, Cochran’s *Q* and *I*^2^ were reported. Large heterogeneity was determined as *I*^2^ > 50% [36]. Cook’s distances were used to identify influential outliers with values greater than the median plus six times the interquartile range of the Cook’s distances considered influential. Publication bias was assessed using funnel plots. Rank correlation and regression tests using the standard error of the observed outcomes as predictor were used to check for funnel plot asymmetry [37, 38]. Results are presented with 95% confidence intervals (CI). Findings from studies without adequate data for inclusion in the meta-analysis were reported narratively.

## Results

### Overview

**Fig. 1:**
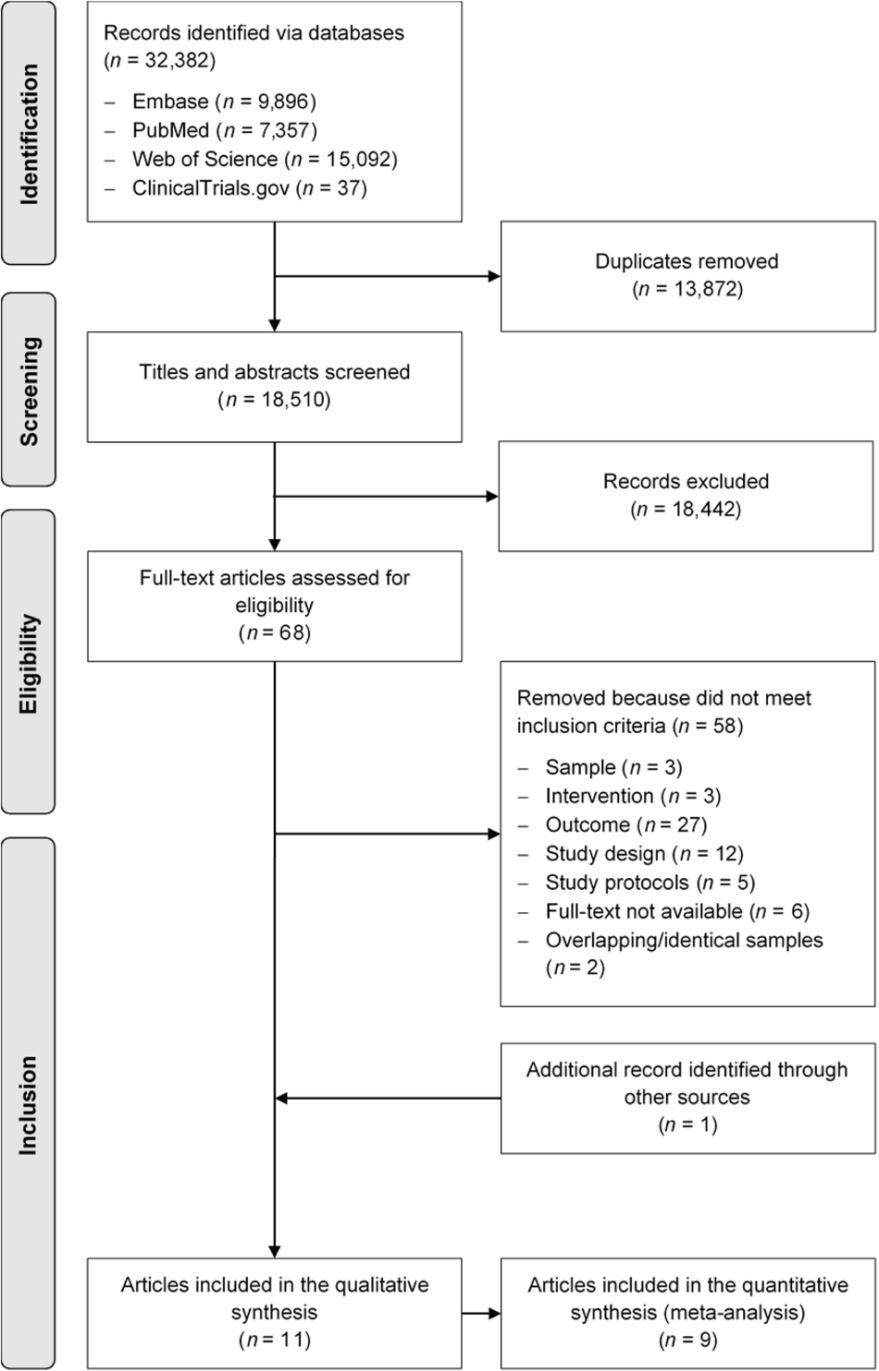
PRISMA flowchart. PRISMA (Preferred Reporting Items for Systematic Reviews and Meta-Analyses) flowchart of the search and selection process for the systematic review and meta-analysis of dietary interventions to improve body composition in men treated with androgen deprivation therapy for prostate cancer.

The results of the literature search are summarised in Fig. 1. The search identified 32,382 articles, of which 13,875 were duplicates and 18,442 were removed after screening of titles and abstracts. Full texts were retrieved and assessed for 68 articles, of which ten were deemed eligible. One additional article was identified through hand searching of reference lists, resulting in a total of eleven articles with data from 536 participants included in this review.

### Study characteristics

The characteristics of the eleven included studies are presented in Supplementary Table 1. All studies were RCTs published from 2009 onwards. Duration of the interventions ranged from 3 to 12 months. Seven studies investigated the effects of a dietary advice intervention [29, 30, 39–43] and four studies included nutritional supplementation [31, 44–46]. Dietary advice consisted of general healthy-eating guidelines in five studies [29, 39, 40, 42, 43], and a low-carbohydrate [30] or low-glycaemic index diet [41] in one study each. Nutritional supplements contained protein powder, whey in two studies [31, 44] and soy in one study [45], and vitamin D in one study [46]. Dietary advice was delivered using various formats, including written general nutrition recommendations or specific instructions, individual or group counselling or a combination of these. Eight studies combined the dietary intervention with a physical activity component, which included supervised exercise in five studies [29, 31, 39, 40, 44] and exercise recommendations in three studies [30, 41, 42]. Supervised exercise protocols included resistance exercise, either alone or combined with aerobic exercise, in all five studies, while studies providing exercise recommendations to participants only included aerobic exercise. The comparator consisted of a usual care group in all studies but two supplementation studies [45, 46], where participants in the comparator group received a placebo. The detailed intervention protocols are outlined in Table 2. Body composition was assessed using DXA in five studies [29–31, 43, 44], bioelectrical impedance analysis in three studies [40, 41, 46] and skinfold thickness measurements in one study [42]. Two studies [39, 45] reported BMI as the only body composition outcome.

**Table 2:** Summary of interventions and dietary intake pre- and post-intervention in the included studies.

References	Intervention protocol		Protocol duration	Comparator	Method of diet assessment	Total energy (kcal/d)		Protein (g/d)	
	Diet	Exercise				Baseline	Endpoint	Baseline	Endpoint
Baguley et al. [43]	Mediterranean diet: <10% total energy from saturated fat, 2 servings/d fruit, 5 servings/d vegetables, 30 g/d fibre, reduce or eliminate red/processed meats, 3 servings/week fish, 2 servings/d dairy, 1 serving/d nuts and seeds, ≤2 units alcohol/week; energy reduction if BMI ≥25 kg*m^−2^. Delivery: face-to-face 30–45-min nutrition consultations with a dietitian every 2 weeks to adjust diet to nutrient requirements and dietary preferences.	None	12 weeks	Usual care	Wollongong Dietary Inventory for the last month of intervention	I: 2462 (339) C: 2328 (342)	I: 2046 (337)^a^ C: 2366 (332)^b^	I: 110 (16) C: 106 (16)	I: 108 (16)^a^ C: 107 (16)^b^
Bourke et al. [39] ^c^Same trial as Gilbert et al. [40] but different sample	Diet: reduction in dietary fat intake to approximately 25% of total energy intake, ≥5 servings/d fruit and vegetables, increased fibre consumption, decreased intake of refined carbohydrates and limiting alcohol intake to 1–2 units/d. Delivery: nutrition advice pack provided to participants, small-group healthy-eating seminars lasting 15–20 min every 2 weeks.	Supervised and self-directed aerobic and resistance exercise	12 weeks	Usual care	3-day food diaries	I: 1957 (457) C: 2012 (623)	I: 1669 (351)^a^ C: 1983 (560)^a,c^	I: 82 (19) C: 80 (29)	I: 73 (15)^a^ C: 75 (17)^a^
Chaplow et al. [29]	Diet: reduction in energy intake by 500–1000 kcal/d; reduction in total fats to 25–30%, saturated fats to 7% and protein to 15% of total calories; 5 servings/d fruit and vegetables; ≥3 servings/d of whole grains and a gradual increase to ≥25 g/d fibre. Delivery: counselling sessions with a registered dietitian (8x group-mediated, 2x via phone calls).	Supervised aerobic and resistance exercise	12 weeks	Written dietary and exercise advice; 20-min phone contact with study staff every 2 weeks	No assessment	Not reported	Not reported	Not reported	Not reported
Dalla Via et al. [31]	Supplement: 25 g/d of whey protein (not specified) containing 2.4 g leucine, plus daily dosis of 1200 mg calcium carbonate and 1000 IU vitamin D. Delivery: daily ingestion in form of powder mixed with water.	Supervised and self-directed aerobic and resistance exercise	52 weeks	Usual care	No assessment	Not reported	Not reported	Not reported	Not reported
Dawson et al. [44]	Supplement: 50 g/d of whey protein isolate (EnergyFirst^®^, Manhattan Beach, CA) containing 225 kcal, 50 g protein (4-g leucine), 0 g fat, 7.5 g carbohydrate. Delivery: daily supplement consumed in 2 doses of 25 g each.	Supervised resistance exercise	12 weeks	Home-based flexibility program	3-day food diary	I: 1976 (708) C: 1561 (284)	I: 2062 (753)^b^ C: 1624 (330)^b^	I: 94 (34) C: 77 (31)	I: 118 (30)^b^ C: 88 (34)^b,c^
Freedland et al. [30]	Low-carbohydrate diet: carbohydrate intake ≤20 g/d; a list of low-carbohydrate foods to choose from, a list of moderate/high carbohydrate foods to limit, sample menus and recipes were provided. Delivery: counselling with a dietitian in person or by phone weekly for months 0–3 and every 2 weeks thereafter.	Instructions to walk ≥30 min/d for ≥5 d/week	24 weeks	Usual care	3-day food diary	I: 2212 (1850, 2616) C: 1728 (1482, 2554)	I: 1698 (1428, 1958)^a^ C: 1633 (1404, 2304)^a^	I: 96 (74, 128) C: 80 (53, 87)	I: 116 (84, 147)^b^ C: 74 (58, 100)^a,c^
Gilbert et al. [40] *Same trial as Bourke et al. [39] but different sample	Diet: reduction in dietary fat intake to approximately 25% of total energy intake, ≥5 servings/d fruit and vegetables, increased fibre consumption, decreased intake of refined carbohydrates and limiting alcohol intake to 1–2 units/d. Delivery: nutrition advice pack provided to participants, small-group healthy-eating seminars lasting 15–20 min every 2 weeks.	Supervised and self-directed aerobic and resistance exercise	12 weeks	Usual care	3-day food diary	I: 1944 (487) C: 2084 (542)	I: 1870 (392)^a^ C: 1931 (554)^a^	I: 81 (29) C: 81 (20)	I: 80 (20)^a^ C: 81 (25)^a^
Inglis et al. [46]	Supplement: high-dose vitamin D3 with 50,000 IU/week plus daily multi-vitamin with 600 IU/d vitamin D, 210 mg/d calcium and 800 mg/d calcium supplements. Delivery: not reported.	None	24 weeks	Low-dose vitamin D3 with placebo plus daily multi-vitamin identical to intervention	No assessment	Not reported	Not reported	Not reported	Not reported
Nobes et al. [41]	Diet: advice for low-glycaemic index diet, no further information specified; plus daily administration of 850–1700 mg metformin. Delivery: comprehensive guidebook.	Individually tailored instructions for regular aerobic exercise	24 weeks	Usual care	Diary of intervention compliance	Not reported	Not reported	Not reported	Not reported
O’Neill et al. [42]	Diet: UK healthy-eating guidelines; ≥5 servings/d vegetables and fruits; 30–35% of total energy from fat, <10% energy from saturated fat; 10% of energy from polyunsaturated fat; 25–35 g/d fibre; ≤28 units/week alcohol; limit processed meats and foods high in salt and/or sugar; calorie reduction for overweight participants. Delivery: individually tailored dietary guidebook, phone contact every 2 weeks for months 0–3 and every 3 weeks thereafter.	Instructions to walk for ≥30 min/d for ≥5 d/week with a pedometer provided to track step counts	24 weeks	Usual care	7-day food diary	I: 2272 (521) C: 2128 (376)	I: 1889 (419)^a^ C: 2017 (476)^a,c^	I: 89 (17) C: 85 (18)	I: 84 (18)^a^ C: 83 (22)^a^
Sharma et al. [45]	Supplement: 20 g/d of soy protein powder (Revival^®^, Physicians Pharmaceuticals, Inc, Kernersville, NC) containing 160 mg of total isoflavones (64 mg genistein, 63 mg daidzein and 34 mg glycitein). Delivery: daily ingestion of powder mixed with beverages.	None	12 weeks	Placebo: 20 g/d whole milk protein powder with similar nutrient content except isoflavones	No assessment	Not reported	Not reported	Not reported	Not reported

Data presented as mean (standard deviation) except for Freedland et al. who reported median (25th percentile, 75th percentile).

*BMI* body mass index, *C* comparator, *d* day, *I* intervention group, *IU* international units, *kcal* kilocalorie, *min* minute(s).

^a^Decrease from baseline to endpoint value.

^b^Increase from baseline to endpoint value.

^c^Significant between-group difference (*p* < 0.05).

### Risk of bias in studies

**Fig. 2:**
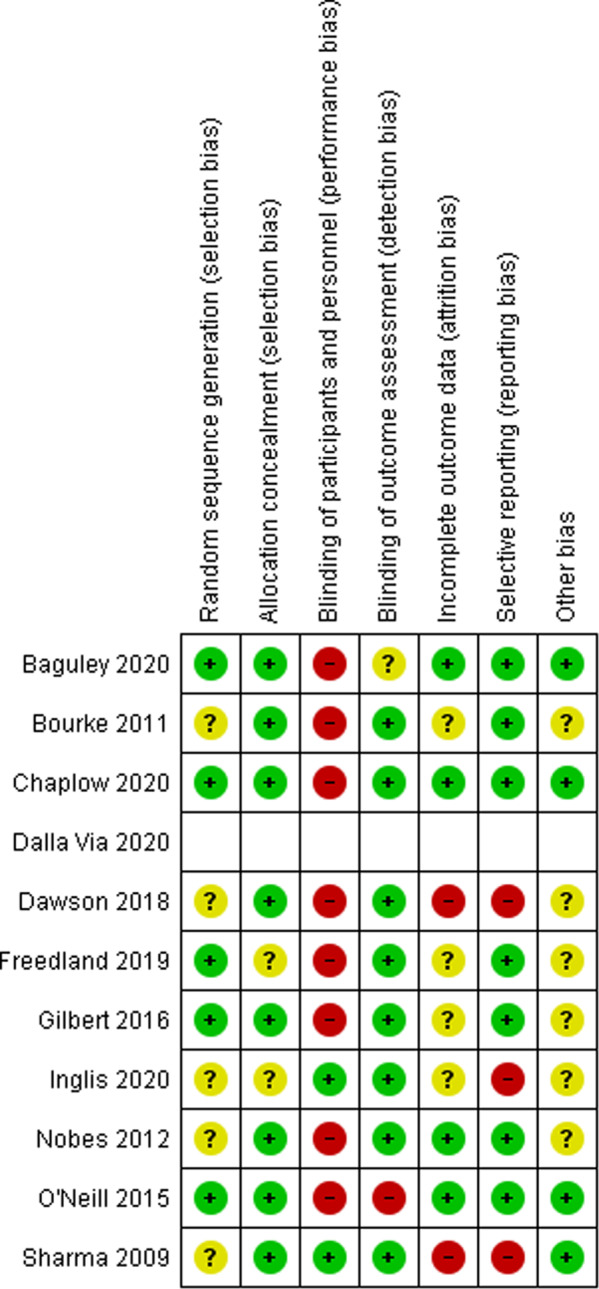
Summary of risk of bias assessment. The methodological quality of the included studies was assessed using the Cochrane Collaboration’s tool for assessing risk of bias. Green indicates a low risk of bias, red indicates a high risk of bias, and yellow indicates an unclear risk of bias.

A summary of the risk of bias in the included studies is shown in Fig. 2. Most studies reported appropriate allocation concealment and blinding of study personnel conducting outcome assessments. The most common sources of methodological bias were lack of blinding of participants and study personnel during the intervention [29, 30, 39–44], selective reporting [44–46] and incomplete reporting of outcome data [44, 45]. The risk of bias of one study [31] published only as an abstract could not be determined.

### Changes in lean mass

**Fig. 3:**
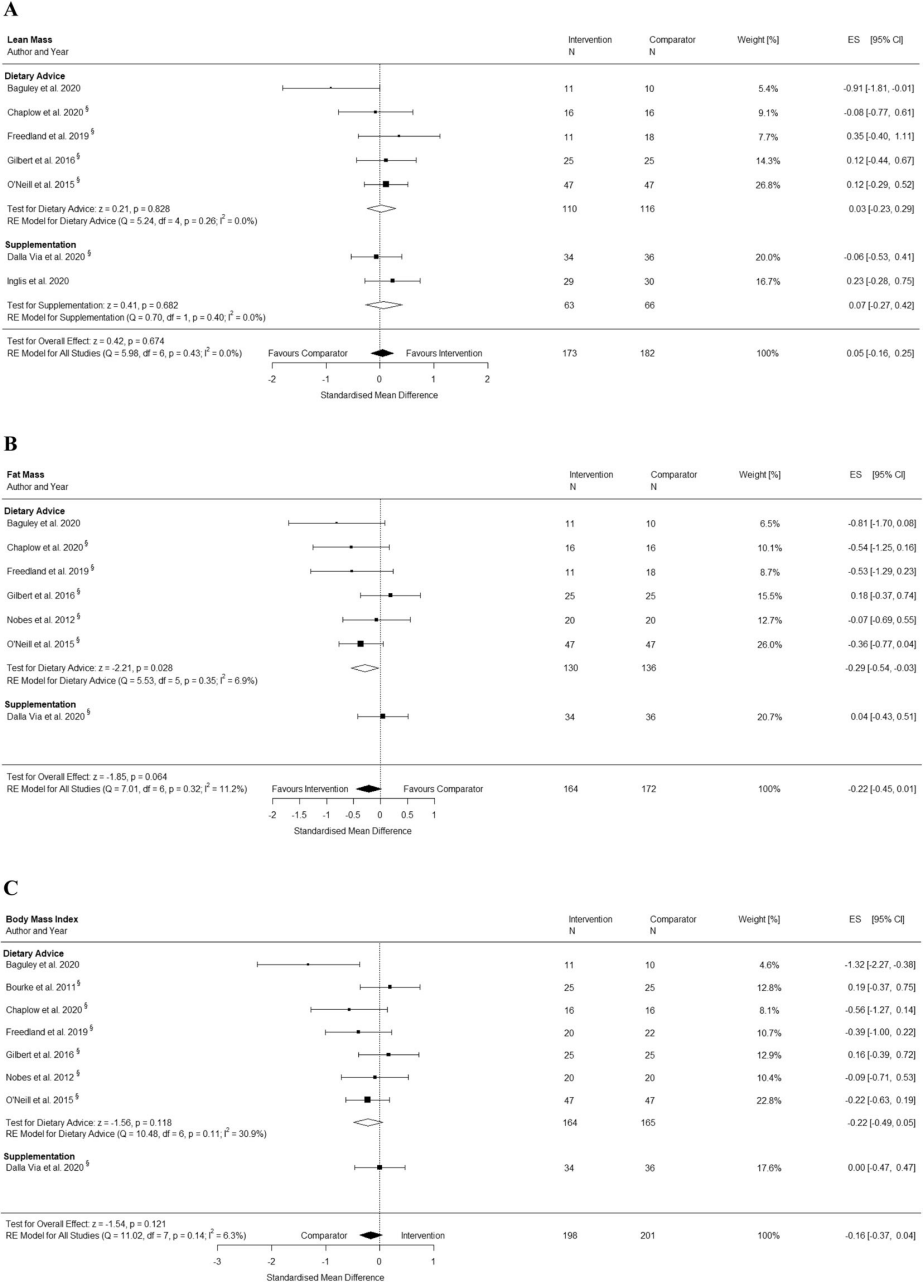
Effects of dietary advice and supplementation interventions. **A** Lean mass, **B** fat mass, and **C** body mass index (BMI) in men treated with ADT for prostate cancer. Forest plots showing the results of meta-analyses of post intervention values for each outcome using a random effects model. Because changes in BMI cannot clearly be defined as favourable for either group, we refrained from using the term ‘favours’. Effects situated left of the middle line present a higher BMI in the comparator group, whereas effects situated right of the middle line present a higher BMI in the intervention group. CI confidence interval, ES effect size, RE random effects.

Seven studies [29–31, 40, 42, 43, 46] including 355 participants reported lean mass pre and post intervention. The results from the pooled analysis showed that dietary interventions did not significantly increase lean mass. The pooled mean difference in total lean mass was 0.05 kg (95% CI: −0.16, 0.25; *p* = 0.674) with low heterogeneity (*I*^2^ = 0%) (Fig. 3A). The exclusion of two supplementation interventions [31, 46] from the pooled analysis did not change the significance of the results.

### Changes in fat mass

Seven studies [29–31, 40–43] including 336 participants reported fat mass pre and post intervention. The results from the pooled analysis showed that dietary interventions did not significantly decrease fat mass. The pooled mean difference in total fat mass was −0.22 kg (95% CI: −0.45, 0.01; *p* = .064) with low heterogeneity (*I*^2^ = 11%) (Fig. 3B). The exclusion of the only supplementation intervention [31] from the pooled analysis resulted in a significant pooled mean difference of −0.29 kg (95% CI: −0.54, −0.03; *p* = .028; *n* = 266) with low heterogeneity (*I*^2^ = 7%) for dietary advice interventions.

### Changes in BMI

Eight studies [29–31, 39–43] including 399 participants reported BMI pre and post intervention. The results from the pooled analysis showed no significant effect of dietary interventions on BMI. The pooled mean difference in BMI was −0.16 kg*m^−2^ (95% CI: −0.37, 0.04; *p* = 0.121) with low heterogeneity (*I*^2^ = 6%) (Fig. 3C). The exclusion of the only supplementation intervention [31] from the pooled analysis did not change the significance of the results.

### Narrative reporting of study results

Two studies [44, 45] on the effects of nutritional supplementation on body composition outcomes did not report post-intervention measures. Sharma et al. [45] examined the effect of daily intake of 20 g soy protein containing 160 mg isoflavones compared to 20 g whole milk powder over 12 weeks. They assessed body composition using BMI, which they report did not change significantly in either group. In addition, a four-armed RCT that investigated the effect of resistance training combined with 50 g whey protein isolate daily over 12 weeks, compared to either of those interventions alone and a comparator group, found that protein supplementation did not influence body composition outcomes [44]. In this study, body composition was assessed using DXA, but post-intervention measures were only reported as pooled data for training and non-training groups.

## Discussion

ADT elicits adverse effects on body composition that include increased body fat accumulation, a concomitant BMI rise and accelerated muscle wasting [47]. Because ADT causes such drastic metabolic alterations, dietary interventions have been proposed as a way to mitigate treatment-related side effects. The evidence on the potential benefits of such interventions is limited though. We present the first meta-analysis of prospectively collected data on the effects of dietary interventions on body composition outcomes in men treated with ADT for prostate cancer. Eleven RCTs that reported the effects of dietary advice or nutritional supplementation on lean mass, fat mass and BMI were identified. The results from our meta-analysis show that interventions using dietary advice have the potential to reduce fat mass in men treated with ADT, whereas lean mass and BMI remain mostly unaltered irrespective of the intervention type. However, the effect on fat mass did not persist when supplementation studies were added to the analysis, which may be due to the considerable heterogeneity of intervention designs and aims.

Loss of lean mass is a metabolic alteration commonly observed in patients with cancer and can be caused by the tumour, treatments, modified diet or physical inactivity [20, 48]. There is consistent evidence that low muscle mass is associated with declines in physical function and quality of life, increased frailty and a higher mortality risk [20, 49]. Treatment with ADT augments these catabolic processes due to the inhibition of androgen signalling, which plays a critical role in the regulation of muscle protein synthesis, and thereby exacerbates ageing-related muscle wasting [13]. Our results indicate that none of the dietary interventions reversed this process by increasing lean mass. Considering the adverse metabolic state induced by ADT and the loss of lean mass reported by Galvão et al. [5], simply preserving pre-intervention values of lean mass may already be a success. Preservation of lean mass while achieving fat mass loss was only reported by one intervention, which combined dietary and walking advice [42], but this finding may have been influenced by the outcome measure as they reported relative lean mass in contrast to absolute lean mass in the other included studies. Low protein intake, which is frequently found among patients with cancer, can further contribute to the loss of skeletal muscle induced by ADT. Current guidelines on nutrition for patients with cancer recommend a daily protein intake of 1–1.5 g/kg bodyweight to preserve lean mass [20]. We examined the changes in dietary intake in all six studies that reported diet assessment outcomes (see Table 2) and found that only two interventions, a low-carbohydrate diet [30] and protein supplementation [44], were associated with increased protein intake, while all interventions reported reductions in total energy intake except for the aforementioned protein supplementation [44]. A substantial reduction in total energy intake while failing to increase dietary protein intake may promote loss of lean mass. Such a trend was prevalent in one study [43], which reported a 2.5% reduction in lean mass after 12 weeks of a Mediterranean-style diet aimed at decreasing dietary fat intake and increasing intake of fruits, vegetables and fibre. The authors argue that the dietary protein intake may have been insufficient to preserve lean mass during intentional weight loss.

Nutrient deficiencies of patients with cancer may be caused by high energy demands of the tumour or impeded gastrointestinal uptake, and supplementation of specific nutrients has been suggested as a potential treatment [20, 24]. Despite our comprehensive search, we identified only two studies [31, 44] that supplemented dietary protein and provided lean mass measures. Dawson et al. [44] did not observe an effect of daily supplementation of 50 g whey protein isolate, divided into two doses, on lean mass over 12 weeks with or without supervised resistance exercise, however, post-intervention measures for each group were not provided. While the average daily protein intake during the intervention was significantly higher in the supplementation groups, the supplementation only group nevertheless had the lowest dietary protein to bodyweight ratio of all groups. It is unclear whether the data were skewed by one participant in this group, who reportedly refused to take the supplement after 2 weeks but was still included in the analysis. The net protein increase in the intervention groups was less than half of the supplemented 50 g per day, highlighting the need for frequent diet assessments to avoid unintentional changes in the habitual diet of the participants. Interestingly, the combined supplementation and exercise group, which showed the highest dietary protein intake with 1.4 g/kg bodyweight, consumed one of the two daily doses immediately after the supervised exercise sessions, presumably increasing compliance with the intervention. It is worth noting that, despite evidence that protein supplementation after resistance exercise evokes an acute muscle protein synthesis response in men on ADT [50], the only study with such a design that could be included in the meta-analysis was by Dalla Via et al. [31]. They observed no significant changes in lean mass in participants of a 12-month intervention that combined daily supplementation of whey protein, calcium carbonate and vitamin D with supervised resistance exercise. Participants were also instructed to consume the supplement within 2 h post-exercise on training days to increase compliance, but whether the daily dose of 25 g protein was sufficient to reach the recommended intake level remains unclear because diet assessment were not reported. Despite the lack of significant effects, the protocols used by both studies present promising approaches as concomitant resistance exercise has been shown to enhance the stimulatory effect of amino acid intake on muscle protein synthesis [24], a physiological mechanism not aided by any of the other interventions. In addition, Inglis et al. [46] reported a significant lean mass increase following 12 weeks of high-dose vitamin D supplementation, but the effect did not persist after 24 weeks. Evidence from patients with advanced cancer of various types shows that supplementation of the amino acid-related nutrients beta-hydroxy-beta-methylbutyrate, arginine and glutamine was associated with a significant lean mass increase after only 4 weeks compared to patients who received a supplement containing non-essential amino acids [51]. Altogether, protein supplementation could help to balance deficits, but timing, quantity and composition of the supplement may be crucial and future studies should investigate whether this approach would counteract the chronic effects of ADT on muscle physiology.

Regarding fat mass, our findings show a beneficial effect of the dietary interventions with significant reductions reported in four studies [29, 30, 41, 42], while three studies observed no differences between intervention and comparator groups [31, 40, 43]. None of the studies reported fat mass gains in the intervention groups but in several comparator groups. The pooled analysis showed that dietary advice interventions were associated with a significant fat mass reduction but the effect was no longer present when the results from Dalla Via et al. [31], the only protein supplementation intervention that measured fat mass, were included. This may be explained by considerable heterogeneity among the intervention designs. Because weight gain is a common side effect of ADT [47], most dietary advice interventions focused on calorie restriction to achieve a negative energy balance, whereas none of the supplementation interventions aimed to change total energy intake. In fact, all studies reported a baseline BMI above the 25 kg*m^−2^ cut-off for overweight [52]. This puts men treated with ADT at an increased risk for obesity, metabolic syndrome, cardiovascular disease and frailty [53]. Chaplow et al. [29] reported that fat mass reduction was associated with improved mobility performance following a combined diet and exercise intervention. Overall, these findings highlight the promising potential of interventions that promote changes in dietary behaviour such as reducing calories to mitigate ADT-related side effects, irrespective of their effect on lean mass.

Despite changes in fat mass in some studies, we observed no differences in BMI between groups irrespective of the intervention type. Among the studies not included in our meta-analysis, Sharma et al. [45] administered a soy protein supplement containing isoflavones, which are known for their phytoestrogenic effects, but did not affect BMI. Because the comparator group received milk protein, the lack of a between-group effect may be explained by a similar nutrient content of both supplements. Also, BMI as an outcome measure is inadequate to capture potential physiological changes that may have been induced by the supplements, because it neglects the distribution of tissues, such as muscle and fat mass, that differ in their relationship to cancer prognosis [49]. The typical changes in body composition associated with ADT, as well as those intended by diet and exercise interventions, may in fact result in a constant BMI despite substantial changes of total lean and fat mass. Therefore, researchers should use measures that quantify tissue distribution such as DXA, which is considered the ideal method for patients with cancer [54].

Knowledge of ADT-related side effects among men with prostate cancer is lacking, with a study revealing that 65% of men who recently started ADT were unaware that muscle wasting may occur [55]. This lack of information prevents men from engaging in beneficial behaviours, such as regular exercise or a healthy diet, and puts interventions that address these issues into focus. It is well established that by modifying both energy intake and expenditure, interventions that combine exercise and diet generally achieve better weight management results than either of those alone [56]. Exercise protocols of included studies ranged from supervised exercise, alone or combined with aerobic exercise, to walking recommendations, and three studies included no exercise. Resistance exercise in particular has been shown to positively affect body composition and muscle strength in men treated with ADT [53], while considered safe and feasible even for patients with bone metastases [57], which supports the argument to include exercise protocols in future trials. We are aware of a number of ongoing trials, including combined dietary and exercise advice interventions [58, 59], creatine supplementation with resistance exercise [60], and beta-hydroxy-methylbutyrate supplementation (NCT01607879). Future studies should explore options for individualised diet interventions that match the dietary advice or supplement to the nutritional requirements and deficits of the patient, and monitor dietary intake frequently to allow for adjustments if needed.

The strengths of this review include the comprehensive search, which was performed using broad search terms that would encompass all potentially relevant articles. Only prospective, controlled trials with measurements both at baseline and post-intervention were included. The results of this review are limited by the heterogeneity of dietary intervention designs and methods used for body composition assessment. Treatment duration with ADT has been shown to influence both the rate and the total loss of lean mass [15, 47], yet time on ADT at enrolment was not reported for all studies. Most studies included men with a minimum of 3 months on ADT except for one [41], which included hormone-naïve men due to receive ADT and administered the treatment as part of the study. This study, however, did not report lean mass, and changes in fat mass and BMI in the comparator group were similar to other studies, therefore we argue that treatment duration did likely not affect the results. Also, the small number of eligible studies did not allow for subgroup analyses, though neither the rank correlation nor the regression test indicated any funnel plot asymmetry. In addition, not all studies monitored dietary intake or compliance with the diet intervention, limiting the conclusions to be drawn as it remains unclear to which extent diet was modified. Furthermore, the results from two out of three studies that investigated protein supplementation, which is of particular interest for patients with potential nutrient deficiencies, were not included in the meta-analyses due to insufficient reporting of outcome data [44, 45].

## Conclusions

Dietary interventions have the potential to mitigate the adverse changes in lean and fat mass experienced by men treated with ADT. This systematic review and meta-analysis summarises the current body of evidence on the effect of dietary interventions on body composition outcomes. While our results show that dietary advice interventions successfully reduced body fat, the benefits for lean mass were less pronounced. Additional protein supplementation may be required to preserve lean mass during intentional body fat reduction. Considering the benefits of increased muscle mass for morbidity and mortality, future studies should investigate the effects of interventions that combine healthy-eating advice with protein supplementation to achieve energy reduction while balancing nutritional deficits to stimulate muscle protein synthesis. Further research should also examine whether additional resistance exercise enhances the effects of dietary interventions.

## Supplementary information

Supplementary Table 1

## Data Availability

The datasets used and/or analysed during the current study are available from the corresponding author on reasonable request.
