# Supplementary material for: Dietary interventions to improve body composition in men treated with androgen deprivation therapy for prostate cancer: a solution for the growing problem?
Source: Prostate Cancer Prostatic Dis. 2021 Jun 30;25(2):149–58. doi: 10.1038/s41391-021-00411-7 (PMC9184277; doi:10.1038/s41391-021-00411-7)
Supplement: Supplementary file 1 — Supplementary Table 1 [file 41391_2021_411_MOESM1_ESM.docx]

**SUPPLEMENT**

**Supplementary Table 1. Summary of characteristics of the included studies.**

| **Author (year)** | **Country** | **Sample size** | **Age** | **Time on ADT at enrolment** | **Study arms** | **Protocol duration** | **Dropouts** | **Relevant outcomes** | **Method of LM/FM measurement** | **Findings** |
| --- | --- | --- | --- | --- | --- | --- | --- | --- | --- | --- |
| Baguley et al. (2020)  Baguley et al. (2017) | Australia | N = 23  I: 12  C: 11 | I: 66.6 y (7.6)  C: 65.1 y (7.9) | I: 36.4 months (38.3)  C: 31.0 months (32.2) | I: Mediterranean diet advice  C: usual care | 12 weeks | n = 2  I: 1  C: 1 | LM  FM  BMI | DXA | LM, FM: No differences  BMI: *Unpublished data*^a^ |
| Bourke et al. (2011)*  **same trial as Gilbert et al. (2016) but different sample* | UK | N = 50  I: 25  C: 25 | I: 71.3 y (6.4)  C: 72.2 y (7.7) | I: 30 months (31)  C: 30 months (31) | I: lifestyle intervention (dietary advice with supervised and self-directed exercise)  C: usual care | 12 weeks | n = 7  I: 4  C: 3 | BMI | - | BMI: No differences |
| Chaplow et al. (2020)  Focht et al. (2018) | USA | N = 32  I: 16  C: 16 | I: 67.9 y (7.9)  C: 64.3 y (6.1) | I: 26.0 months (25.5)  C: 20.4 months (21.0) | I: exercise and dietary behaviour change intervention (dietary advice with supervised exercise)  C: usual care | 12 weeks | n = 7  I: 2  C: 5 | LM  FM  BMI | DXA | Significant intervention effect on FM (d = ‒0.86; p = .022), but not LM (p > .05).  BMI: *Unpublished data*^a^ |
| Dalla Via et al. (2020)  Owen et al. (2017) | Australia | N = 70  I: 34  C: 36 | Not reported | Not reported | I: daily protein supplementation and supervised exercise  C: usual care | 52 weeks | n = 12  I: 5  C: 7 | LM  FM BMI | DXA | LM, FM: No differences  BMI: *Unpublished data*^a^ |
| Dawson et al. (2018)  Kiwata et al. (2017) | USA | N = 37  I1: 8  I2: 10  C: 11 | Not reported | Not reported | I1: daily protein supplementation and supervised resistance exercise  I2: daily protein supplementation  C: unsupervised stretching | 12 weeks | n = 4  I1: 2  C: 2 | LM  FM | DXA | No significant main effect of protein or interaction effect of protein + exercise was observed for any of the body composition variables (p > .05). |
| Freedland et al. (2019) | USA | N = 42  I: 20  C: 22 | I: 66 y (61, 76)^b^  C: 66 y (56, 70)^b^ | Not reported | I: low-carbohydrate diet and walking advice  C: usual care | 24 weeks | n = 13  I: 9  C: 4 | LM  FM  BMI | DXA | LM, FM: Greater decrease in LM (-7.3%, p = .036) and FM (-16.2%, p = .002) in intervention group.^a^  BMI: Significant reduction in intervention group, while comparator group showed increase (p < .001).^a^ |
| Gilbert et al. (2016)  **same trial as Bourke et al. (2011) but different sample* | UK | N = 50  I: 25  C: 25 | I: 70.1 y (5.3)  C: 70.4 y (9.2) | I: 19 months (12, 95)^c^  C: 18 months (6, 92)^c^ | I: lifestyle intervention (dietary advice with supervised and self-directed exercise)  C: usual care | 12 weeks | n = 8  I: 3  C: 5 | MM  FM  BMI | BIA | MM: Significantly higher MM in the intervention group at 12 weeks (p = .03), but both groups showed a similar increase from baseline.  FM, BMI: No differences |
| Inglis et al. (2020) | USA | N = 59  I: 29 C: 30 | I: 67.5 y (6.4)  C: 67.8 y (4.4) | I: 39.7 weeks (37.6)  C: 47.9 weeks (37.2) | I: daily high-dose vitamin D supplementation  C: placebo (low-dose vitamin D) | 24 weeks | n = 0 | LM | BIA | LM: No differences |
| Nobes et al. (2012) | UK | N = 40  I: 20  C: 20 | I: 70.5 y (58 – 80)^c^  C: 69.5 y (56 – 84)^c^ | Not reported | I: low glycaemic index diet and exercise advice plus daily metformin  C: usual care | 24 weeks | n = 0 | FM  BMI | BIA | FM: No differences  BMI: Significant reduction in intervention group, while comparator group showed increase (p < .001). |
| O’Neill et al. (2015) | UK | N = 94  I: 47  C: 47 | I: 69.7 y (6.8)  C: 69.9 y (7.0) | I: 26.4 months (32.4)  C: 19.3 months (18.6) | I: dietary and walking advice  C: usual care | 24 weeks | n = 4  I: 2  C: 2 | LM  FM  BMI | Skinfold thickness | Significant intervention effect on FM (p < .001) and BMI (p < .001), but not LM (p > .05). |
| Sharma et al. (2009)  Napora et al. (2011) | USA | N = 39  I: 20  C: 19 | I: 69.2 y (2.5)^d^  C: 69.0 y (2.2)^d^ | I: 2.4 y (0.4)^d^  C: 2.0 y (0.6)^d^ | I: daily soy protein supplementation  C: placebo (milk protein) | 12 weeks | n = 6  I: 3  C: 3 | BMI | - | BMI: No differences |

*Notes*: Sample sizes refer to the total number of randomised patients. Data presented as mean (standard deviation) if not indicated otherwise.

BIA: bioelectrical impedance analysis; BMI: body mass index; C: control group; CI: Confidence interval; DXA: dual-energy X-ray absorptiometry; FM: fat mass; I: intervention group; LM: lean mass; MM: muscle mass; UK: United Kingdom; USA: United States of America; y: years.

^a^ Unpublished data provided by the study authors is not reported under findings, but was included in the analysis.

^b^ Data presented as median (25^th^ percentile, 75^th^ percentile).

^c^ Data presented as median (range).

^d^ Data presented as mean (standard error).
